# A 3D geomodel of the deep aquifers in the Orléans area of the southern Paris Basin (France)

**DOI:** 10.1038/s41597-022-01876-4

**Published:** 2022-12-24

**Authors:** Perrine Mas, Philippe Calcagno, Séverine Caritg-Monnot, Laurent Beccaletto, Laure Capar, Virginie Hamm

**Affiliations:** 1 Bureau de Recherches Géologiques et Minières (BRGM), Orléans, France; 2 Géosciences Paris-Saclay, Université Paris-Saclay, CNRS, Orsay, France

**Keywords:** Geology, Geophysics

## Abstract

An increasing number of cities are interested in deep geothermal energy in order to increase the share of renewable energies in their district heating networks. To reduce the risks related to deep geothermal energy operations, reliable digital models are needed: they make it possible to predict the depth of aquifers away from borehole locations, and their thermal and hydrological evolution by supporting detailed water and heat flow simulations. This paper presents a 3D geomodel developed for this purpose in the southern Paris Basin of France in the Orléans area. The 3D geomodel integrates various data such as reprocessed and interpreted seismic lines, well data, and a pre-existing larger-scale and lower-resolution 3D geological model. The resulting 3D geomodel gives a new and reliable representation of the main aquifers underlying the study area. Within the framework of the project, hydrological and thermal simulations were then performed based on this 3D geomodel. Other environmental investigations (e.g. CO_2_ storage) and teaching/communication activities could also benefit from the dataset.

## Abstract (summary)

**Table:** 

Measurement(s)	geological features
Technology Type(s)	3D geomodelling integration
Sample Characteristic - Location	Southern Paris Basin (France)

## Background & Summary

Three-dimensional geological and reservoir models have become a very common tool in many applications in earth sciences over recent decades^1,2^. They are used to better understand the geology of an area by constructing a coherent interpretation of the structures and by merging 2D data in a 3D space. In the underground exploration, engineering or management industries, geological models are the basis for calculations, such as predictive fluid flow simulations and resource evaluations^1,3–5^. Therefore, geological models are currently commonly used in, among other domains: applications related to geotechnics^6^, hydrogeology^7,8^, seismic hazard assessments^9^, energy resources^10,11^ (e.g. geothermal energy and hydrocarbons), and underground storage^12^ (e.g. heat, gas, CO_2_, and waste). In addition, geological models are also really useful for communication and educational purposes by visualizing 3D subsurface information^5,13^.

Geothermal development in the Paris Basin started in the early-1970s in reaction to the first oil crisis^14^. After a period of withdrawal, there was a boost of activity at the end of the 1990s following the Kyoto Agreement, and the “Geothermal Energy in Ile-de-France Revival Program” in the late 2000s^14,15^. This region presently includes 50 out of the more than 70 deep geothermal energy installations dedicated to urban heating in France, most of which target the Dogger (middle Jurassic) aquifer. The installations are mostly “doublets” which comprise a production and an injection well, forming an open loop system^15^. One of the major issues in geothermal energy in the Paris Basin is the “thermal breakthrough”, which is the arrival of the low temperature front from the injector well^15^. South-east of the Paris Basin, the doublet installations are located quite close to one another, increasing the risk of thermal breakthrough induced by another operation. In some areas of the Paris Basin (e.g. south-east of Paris) targeting the Dogger aquifers leads its exploitation to saturation at these places: another installation cannot be implanted without being affected by a thermal breakthrough caused by another doublet. Therefore, the prospection of other underlying or overlying aquifers has begun to further develop geothermal energy in these areas. On an other hand, since the beginning of the 2000’s a secondary interest for the aquifers targeted by geothermal energy has appeared to develop CO_2_ storage: studies targeting the carbonate Dogger formation and the saline aquifers in Triassic silico-clastic formations have been conducted with a view to eventually create a “geological carbon sink”^16,17^. The development of such aquifer exploitation projects (geothermal energy, CO_2_ storage…), sometimes geographically close, reinforces the need to have the most reliable geological models possible.

In response to this challenge, a 3D geomodel was built using the BRGM’s GeoModeller software (see the “Code Availability” section) and as part of a collaboration between two interdisciplinary projects. It was called the Orléans Métropole geomodel (thereafter “OM3D geomodel”) and is located in the southern Paris basin of France in the Orléans area.

The aim of the first project (called “Orléans Métropole”) was to study and assess the deep geothermal potential of the subsurface beneath the metropolis of Orléans in the Centre-Val de Loire French administrative region. The goal of the second project (called “GEOCO2”^18^) was to assess the feasibility of an innovative method of dissolved CO_2_ storage in aquifers^16^ in the same region. The OM3D geomodel was part of the exploratory phase for both projects. Using well and seismic data, it aimed to better characterise the reservoirs and to reduce economical risk of the operations. Its purpose was then to obtain a local-scale, updated and reliable 3D geomodel of the vicinity of Orléans, representing a step forward compared to a pre-existing larger-scale lower-resolution 3D geological model (the SIGES model, see Table 1) covering the entire Centre-Val de Loire region^19^.

**Table 1:** Datasets used as input during the development of the OM3D gemodel.

Type	Name	Original Operator/Institute	Acquisition/Issue date	Notes
Seismic lines	Chartres	Petroleum Exploration Company (CEP)	acquisition: 1963	Selected lines: CH09, CH11, CH12 and CH13
			reprocessing: 2008	
	Loury-Villeny	EURAFREP	acquisition: 1964	Selected lines: LV03, LV09 and LV14
			reprocessing: 2020	
	Loiret 1982/Loiret 1983	Esso	acquisition: 1982–1984	Selected lines: LOIR1, LOIR8, LOIR9, LOIR10, LOIR15, LOIR16, LOIR17, LOIR18, LOIR19, LOIR22
			reprocessing: 2020	
Geological maps	BD Charm-50®	BRGM (French Geological Survey)	2005	1/50 000 geological map of the Loiret department and (for identification of the formation contours)
	BD Million-Géol®		2006	1/1 000 000 geological of the French metropolitan territory (for the fault traces)
				The geological maps were harmonized, simplified and the Sennely fault trace was modified after Beccaletto *et al*., 2008
Digital Terrain Model (DTM)	BD ALTI®	National Institute of Geographic and Forest Information (IGN)	2018	Spatial resolution 25 m
Well data	Marcilly-en-Vilette 1	FROPEX	1959	Petroleum exploration well
				Reaches the Portlandian (TVD = 585 m)
				no velocity survey
	Saint-Sigismond 1	SAFREP	1963	Petroleum exploration well
				Reaches the Basement (TVD = 1299 m)
				velocity survey recorded
	Rebréchien 1	Petroleum Exploration Company (CEP)	1964	Petroleum exploration well
				Reaches the Basement (TVD = 1518 m)
				velocity survey recorded
	Melleray 1	BRGM (French Geological Survey)	1979	Geothermal well
				Reaches the Basement (TVD = 1668 m)
				no velocity survey
	Melleray 2	BRGM (French Geological Survey)	1980	Geothermal well (deviated from 890 m)
				Reaches the Basement (TVD = 1161 m)
				no velocity survey
Pre-existing larger-scale lower-resolution 3D geological model	SIGES Centre Val-de-Loire 3D geological model	BRGM (French Geological Survey)	2019	Mesh resolution: 500 m
				It is composed of surfaces displaying the tops of the main aquifers in the Centre Val de Loire region

TVD = True Vertical Depth (of the Bottom Hole)

The target aquifers for the “Orléans Métropole” and “GEOCO2” projects are the Triassic Sandstones and Dogger (Bajocian-Bathonian) Limestones^20^, and the overlying Tithonian and Lusitanian (Oxfordian) Limestones^21^, respectively. The OM3D geomodel was specifically built to provide the best possible representation of the geometry of these deep aquifers. The aim of this paper is to share the OM3D geomodel so that it can be reused, refined, or modified irrespective of the application domain. Within the “Orléans Métropole” project, the OM3D geomodel was used as a basis for hydrothermal simulations and ultimately for the assessment of the geothermal potential^22,23^. Within the “GEOCO2” project it was used to study the feasibility of CO_2_ storage^21^. However, it could also be used for future applications, e.g. it could be a tool for groundwater management, or energy resources and waste storage for the Orléans metropolis, or as a communication and teaching tool for local representatives and geosciences students. In addition to sharing the geomodel, another objective of this paper is also to provide a methodology that can be applied to other case studies where similar types of data are available^24,25^.

**Fig. 1:**
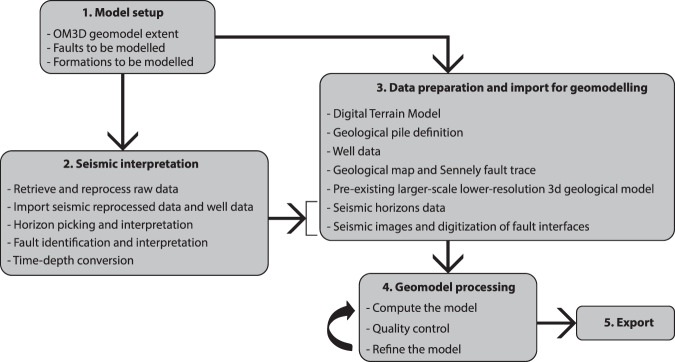
Workflow followed to develop the OM3D geomodel.

The workflow followed to create the OM3D geomodel is shown in Fig. 1. The core steps are: (1) model setup, (2) seismic interpretation, (3) data preparation and import for geomodelling, (4) 3D geomodel processing, including the iterative process of quality control and adjustments which mostly involved comparing the OM3D geomodel to the pre-existing larger-scale lower-resolution 3D geological model, and (5) OM3D geomodel export. These core steps are developed in the “Methods” section.

## Geological and Hydrogeological Contexts

**Fig. 2:**
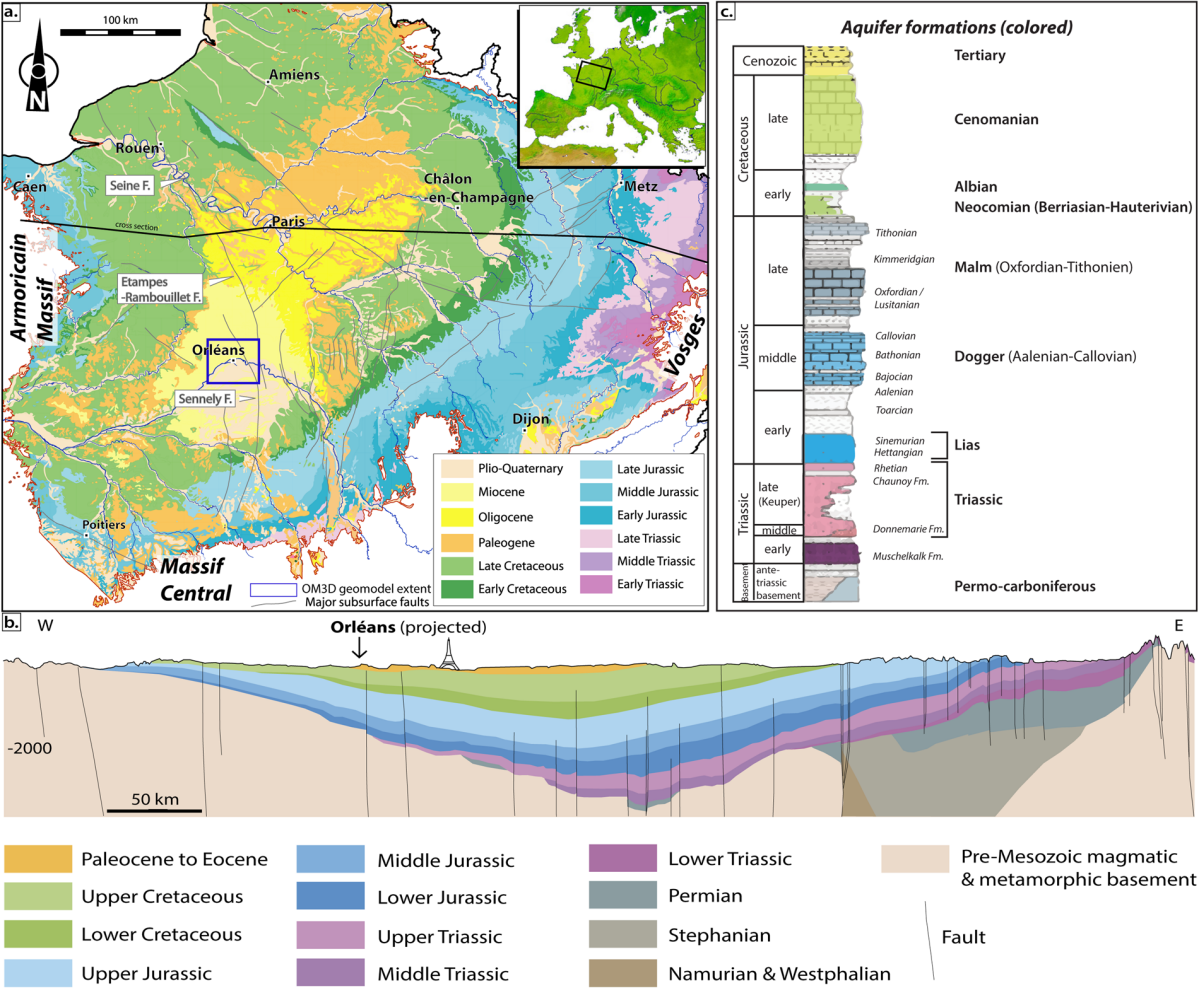
(**a**) Geological overview of the Paris Basin featuring the major subsurface faults. The faults are compiled from Héritier and Villemin^38^, Mégnien and Mégnien^26^, Perrodon and Zabek^33^ and Delmas *et al*.^32^), redrawn and modified by Beccaletto *et al*.^40^. The geological background is from the 1:1 000 000 geological map of France (Chantraine *et al*.). Top-right inset: the Paris Basin in Western Europe. (**b**) Cross section modified after Gély *et al*. (**c**) The stratigraphic log of the Paris Basin with the major aquifer (colored) and aquitard (uncolored) formations is modified after Delmas *et al*.^32^.

**Fig. 3:**
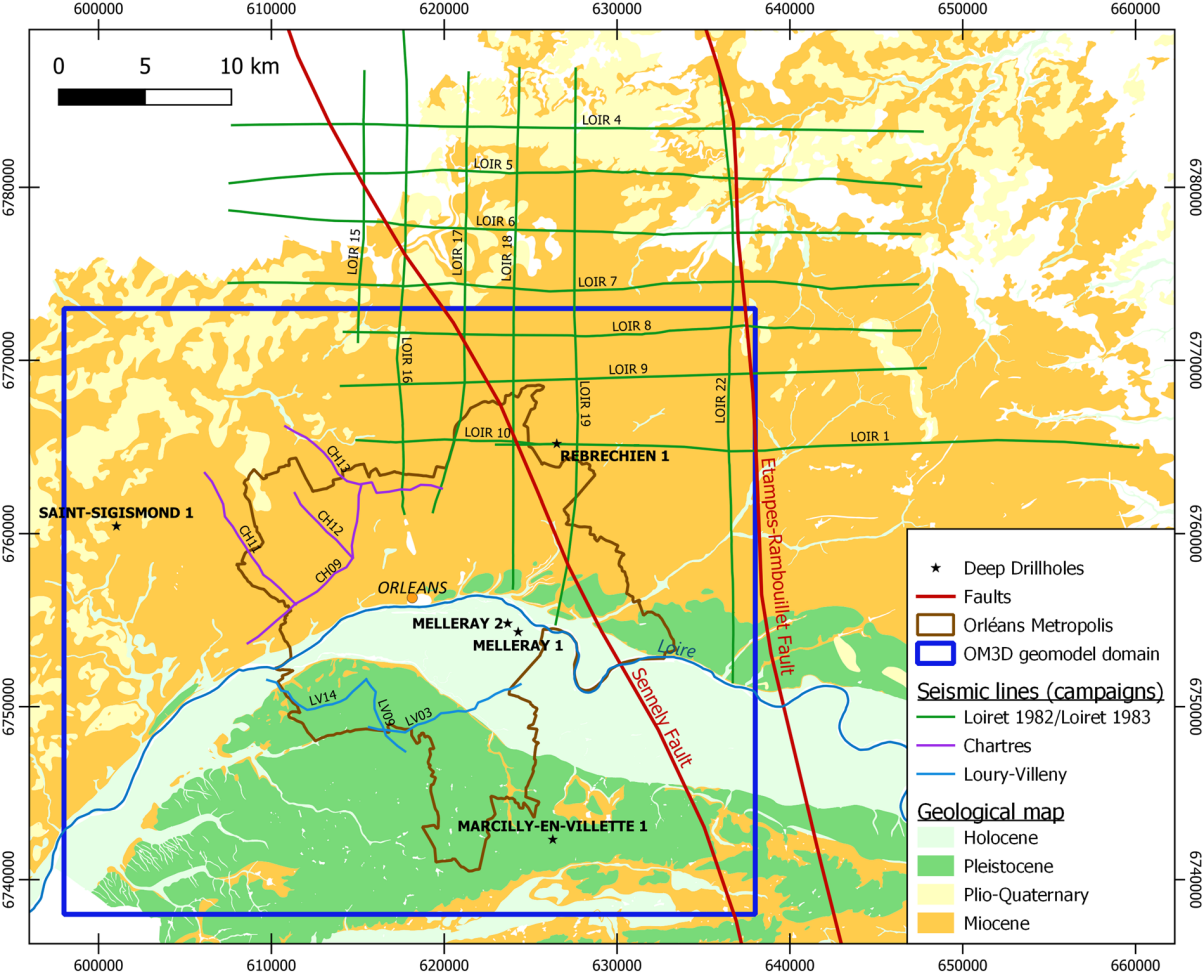
Location of the different input datasets for the development of the OM3D geomodel. The geological background is modified from the 1:50 000 geological map of the Loiret campaign (BD CHARM-50^42^, BRGM). The RGF93/Lambert-93 coordinate system is used.

The Meso-Cenozoic Paris Basin is an intracratonic sedimentary basin lying unconformably over a Paleozoic Variscan basement^26,27^. Both the sedimentary (from open marine to fluvial-continental environments through evaporitic systems, with subsequent major erosional unconformities) and structural (fault activity, long- to short- wavelength folding) patterns successively record the Mesozoic opening of the Alpine Tethys, Atlantic Ocean and Bay of Biscay, and Late Cretaceous-Cenozoic Pyrenean and Alpine orogenies^28–32^. This long-term geological evolution results in the occurrence of major aquifers (Fig. 2) and faults (Fig. 3) in the southwestern part of the Paris Basin and Orléans area.

### Stratigraphy and major aquifers in the orléans area

From the Triassic to the Early Cretaceous, the Paris Basin underwent an overall extensional tectonic regime. Triassic deposits are made of alluvial fan sediments at their base, gradually changing to the Keuper Sandstones (Middle to Late Triassic). The latter are composed of two fluvial bodies separated by a clayey interval^33^: the Donnemarie Sandstones lower body, and the Chaunoy Sandstones upper one. The former is made of proximal to median alluvial fan-type coarse sandstones and conglomerate deposits, whereas the latter is composed of finer sandstones, characteristic of alluvial fans to braided channel systems^34^. The Keuper Sandstones are the major aquifer formation within the Triassic^20^ (Fig. 2). The uppermost Triassic consists of marls and dolomites, which continue to the Lias (Early Jurassic), where an alternation of clays or marls with clayey and often dolomitic limestones are found^29^.

The Toarcian recorded the maximal flooding period, made of open marine deposits such as marls or shales with shell debris, crinoids, or gastropods. This aquitard level corresponds to the “Schistes Carton” also found further to the east of the basin^29^.

The Dogger is divided into three units (Fig. 2): (1) The Aalenian, represented by grey marl containing bioclastic elements and possibly alternating shaly limestone. The proportion is inverted in the lower-Bajocian, formed by shaly limestones with shaly interbeds; (2) The upper Bajocian and the Bathonian, composing the main water-productive levels and mostly consisting of oolitic, gravelly and bioclastic limestones; their light color gave them the name of “Oolithe Blanche”; (3) The overlying Callovian deposits, made of thinner oolitic limestones or marls with bioclastic fragments, and containing a stratigraphic reference level of ferruginous oolites found everywhere in the Paris Basin^33^.

The two main aquifer formations of the Late Jurassic (Malm) are as follows (Fig. 2): (1) the middle and late Oxfordian (Lusitanian), which is composed of a muddy progradational carbonate platform^35^ bounded by the impermeable marls of the early Oxfordian at the base, and the Kimmeridgian at the top^36^, and (2) the Tithonian limestones recording an aggradational muddy carbonate platform ending with coastal plain evaporites^29,35^.

The Lower Cretaceous marks the end of the extensive phase of the basin. The uplift of the basin gave birth to the sandy sediments of the Neocomian and Albian aquifers, deposited over the tilted and truncated Jurassic deposits^26,35^ (Fig. 2). The sea level then rose drastically to cover the whole basin again (global Cenomanian transgression), essentially characterized by chalky aquifer deposits^29,37^. Lastly, the Cenozoic records very low subsidence rates leading to thin and laterally varying sedimentary deposits, forming numerous small-scale shallow aquifers.

For this study, a stratigraphic division was employed to better distinguish the main aquifers from their overlying aquitards (Fig. 2). Each major aquifer formation was then treated individually, whereas the aquitard formations were grouped together. It should be noted that this study focused mainly on the deep aquifers that may present an interest for geothermal energy or storage, and therefore the upper Cretaceous and overlying Cenozoic deposits were not modelled.

### Tectonic setting in the orléans area

The Mesozoic deposits of the southwestern part of the Paris Basin are affected by reactivated Variscan faults, separating the center of the basin to the east from the Variscan Armorican massif to the west^27,38,39^ (Fig. 2). These faults belong to the Seine-Etampes-Rambouillet-Sennely fault system, oriented along a N-S axis in the studied area, turning to a NNW trend in its northern section. They were initiated as strike-slip faults at the end of the Variscan orogeny in Carboniferous times, then reactivated as normal faults during the Permian at the time of the collapse of the Hercynian chain^39^. During the Meso-Cenozoic, these faults mostly act as normal faults with local minor compressional features^40^.

The Seine, Rambouillet and Etampes faults are arranged in a relay zone, and they form a Y-shape with the Sennely fault to which they connect to the south-east of Orléans (Fig. 2). The Sennely fault is important for the realization of the OM3D geomodel because it crosses through the metropolis’ territory. According to Héritier and Villemin^38^, it is unlikely to be a continuous fault, but rather a network of relayed segmented faults, along a meridian direction, with a slight trend towards the north-west north of Rambouillet. The dip generally strongly inclined towards the north-west; the offsets are smaller in the northern part of the fault occurrence, whereas they can reach 500 m in the southern part^38,40^.

## Input Data

The Paris Basin is among the most extensively studied sedimentary basins in the world^29^. Between the 1950s and 1980s, a large amount of subsurface data was acquired during petroleum exploration, leading to better knowledge of the basin’s geology^32^. Numerous hydrocarbon exploration wells were drilled, and seismic acquisition campaigns were conducted. From the 1970s onwards, tens of geothermal doublets were also drilled.

The input data for the OM3D geomodel are: (1) 17 seismic lines, (2) a geological map with the digitized Sennely fault trace, (3) a Digital Terrain Model (DTM), (4) data from five wells (survey reports with geological logs) and (5) a pre-existing 3D larger-scale and lower-resolution 3D geological model which covers a much wider area than the metropolis’ territory (Fig. 3). These data sources are presented in Table 1.

### Seismic data

As mentioned above, many oil companies carried out seismic acquisition campaigns in the Paris Basin during the 1950–1960s and 1980s. For our study, seismic lines acquired around Orléans were retrieved from the oil companies that carried out the campaigns^41^. These seismic data originate from three different acquisition campaigns: (1) Loiret 1982 and Loiret 1983, (2) Loury-Villeny, and (3) Chartres (Fig. 3). Within these raw data sets, only the closest lines to the Orléans metropolis, and those located near the Sennely fault, were selected for interpretation to reduce processing costs. These lines were reprocessed and reinterpreted using up-to-date techniques to give better results than the original processing carried out decades ago. These reprocessed data are the seismic data that were interpreted (for the deposits’s geometry as well as the structural elements) and used for the geomodelling.

Only the datasets from the Chartres and Loury-Villeny campaigns were reprocessed during the projects because the Loiret 1982/Loiret 1983 datasets were already reprocessed in 2008. Some of the lines are outside of the OM3D geomodel domain (Fig. 3), but they were used to better constrain the Sennely fault (especially its northern part) that crosses all of them.

### Geological map

The geological map displayed in Fig. 3 comes from the harmonization of two BRGM (French Geological Survey) geological map datasets: the BD Charm-50^42^ (1/50 000 vectorized geological map of the Loiret department) and the BD Million-Géol^43^ (1/1 000 000 geological map of the entire French metropolitan territory). The Sennely fault was improved and modified after Beccaletto *et al*.^40^ to provide a more reliable fault interpretation. This new fault line was used in the construction of the geomodel to complement the seismic data where no lines were available.

It can be noted that no aquifer formation outcrops in this area, the groundwater recharge coming from the outcropping aquifer formations in the south-west of the Paris Basin. The consequence is also that the geological map cannot be used to constrain the geometry of the modelled formations at the surface, contrary to the modelled faults: the Sennely fault traced on the geological map is very useful to constrain the fault on the topographic section.

### Digital terrain model (DTM)

The BD ALTI® digital terrain model (DTM), provided by the French National Institute of Geographic and Forest Information^44^ (IGN), is a raster dataset with a horizontal cell resolution of 25 m. A higher resolution was not necessary since the altitude variation of the study area is low (the altitudes range between 80 and 150 m above the sea). It represents the ground surface elevation without vegetation or buildings.

### Well data

Geological information beneath the Orléans area is also constrained by well log information^45^. Five wells, including three petroleum ones and one geothermal doublet, are located relatively close to the metropolis and their survey reports contain lithological logs (Fig. 3). They provide information on the sedimentary formation properties based on both logging and testing, as well as on the depth of the tops of the geological formations. The latter are used to tie seismic data to wells, convert the seismic horizons to depth and they were also directly integrated in the 3D geomodel (Table 2 and Fig. 6).

**Table 2:** Aquifers/aquitards formation tops and floors depths for each well. The RGF93/Lambert-93 coordinate system is used.

WELL	X_L93	Y_L93	Wellhead Elevation (m above sea level)	Total Depth (m)	Top Depth (m)	Floor Depth (m)	AQUIFERS/AQUITARDS
RBH1	626516	6765181	133.5	1518	0	624.5	Undifferenciated Cover
RBH1	626516	6765181	133.5	1518	624.5	725	Tithonian Limestones
RBH1	626516	6765181	133.5	1518	725	832	Kimmeridgian Marls
RBH1	626516	6765181	133.5	1518	832	1124	Lusitanian Limestones
RBH1	626516	6765181	133.5	1518	1124	1166	Callovo-Oxfordian Shale
RBH1	626516	6765181	133.5	1518	1166	1311.5	Dogger Limestones
RBH1	626516	6765181	133.5	1518	1311.5	1352	Toarcian Shale
RBH1	626516	6765181	133.5	1518	1352	1385.8	Liassic Limestones
RBH1	626516	6765181	133.5	1518	1385.8	1392.5	Triassic-Liassic Marls
RBH1	626516	6765181	133.5	1518	1392.5	1494	Triassic Sandstones
RBH1	626516	6765181	133.5	1518	1494	1518	Basement
SG1	601036	6760435	113.8	1299.5	0	450	Undifferenciated Cover
SG1	601036	6760435	113.8	1299.5	450	535	Tithonian Limestones
SG1	601036	6760435	113.8	1299.5	535	631.5	Kimmeridgian Marls
SG1	601036	6760435	113.8	1299.5	631.5	907	Lusitanian Limestones
SG1	601036	6760435	113.8	1299.5	907	971	Callovo-Oxfordian Shale
SG1	601036	6760435	113.8	1299.5	971	1125.5	Dogger Limestones
SG1	601036	6760435	113.8	1299.5	1125.5	1212	Toarcian Shale
SG1	601036	6760435	113.8	1299.5	1212	1221	Liassic Limestones
SG1	601036	6760435	113.8	1299.5	1221	1238.5	Triassic-Liassic Marls
SG1	601036	6760435	113.8	1299.5	1238.5	1285	Triassic Sandstones
SG1	601036	6760435	113.8	1299.5	1285	1299.5	Basement
GMY1	624281	6754320	96	1668.75	0	512	Undifferenciated Cover
GMY1	624281	6754320	96	1668.75	512	612	Tithonian Limestones
GMY1	624281	6754320	96	1668.75	612	730	Kimmeridgian Marls
GMY1	624281	6754320	96	1668.75	730	1027	Lusitanian Limestones
GMY1	624281	6754320	96	1668.75	1027	1063	Callovo-Oxfordian Shale
GMY1	624281	6754320	96	1668.75	1063	1237	Dogger Limestones
GMY1	624281	6754320	96	1668.75	1237	1336	Toarcian Shale
GMY1	624281	6754320	96	1668.75	1336	1410	Liassic Limestones
GMY1	624281	6754320	96	1668.75	1410	1436	Triassic-Liassic Marls
GMY1	624281	6754320	96	1668.75	1436	1618	Triassic Sandstones
GMY1	624281	6754320	96	1668.75	1618	1668.8	Basement
GMY2D	623676	6754815	96	1661	0	512	Undifferenciated Cover
GMY2D	623676	6754815	96	1661	512	608	Tithonian Limestones
GMY2D	623676	6754815	96	1661	608	725	Kimmeridgian Marls
GMY2D	623676	6754815	96	1661	725	1021	Lusitanian Limestones
GMY2D	623676	6754815	96	1661	1021	1061	Callovo-Oxfordian Shale
GMY2D	623676	6754815	96	1661	1061	1226	Dogger Limestones
GMY2D	623676	6754815	96	1661	1226	1326	Toarcian Shale
GMY2D	623676	6754815	96	1661	1326	1391	Liassic Limestones
GMY2D	623676	6754815	96	1661	1391	1418	Triassic-Liassic Marls
GMY2D	623676	6754815	96	1661	1418	1582	Triassic Sandstones
GMY2D	623676	6754815	96	1661	1582	1661	Basement
MV1	626284	6742320	129	585	0	537	Undifferenciated Cover
MV1	626284	6742320	129	585	537	585	Tithonian Limestones

### Pre-existing larger-scale and lower-resolution 3D geological model

A pre-existing larger-scale and lower-resolution 3D geological model exists at the scale of the Centre-Val de Loire region (approximately 40,000 km^2^), also covering the area of Orléans; its cell resolution of 500 m is not accurate enough for a deep geothermal application at the scale of a metropolis. This 3D geological model (SIGES) was initiated in 2012 and modified and completed in 2019, using the GDM-Multilayer BRGM software^19^. It is composed of surfaces representing the tops of the main aquifers (down to the basement) in the Centre-Val de Loire region and was created by interpolating data from 12,941 wells throughout the entire region, but with only five deep wells covering the study area (and no seismic data). This model was mainly used for adjusting the Sennely fault geometry in the south-east of the OM3D geomodel (and the formations top near the boundaries of the model).

## Methods

### Model setup

The OM3D geomodel domain, i.e. the 3D bounding box, was chosen based on literature data^40,42,43^ to meet two imperatives: (1) the OM3D geomodel’s extent must cover the metropolis’ territory, and (2) the OM3D geomodel domain must contain as much input data as possible. The area is a rectangle spanning 40 km by 35 km centered on the metropolis (Fig. 3, blue box). Its coordinates are given in Table 3. The Rambouillet-Etampes fault was not included, as it is far enough from the metropolis and lies on the eastern limit of the OM3D geomodel extent.

**Table 3:** OM3D geomodel domain extent. The RGF93/Lambert-93 coordinate system is used.

	Northing (m)	Easting (m)	Elevation (m above sea level)
Minimum	6 738 000	598 000	−2000
Maximum	6 773 000	638 000	500

The choice of the formations to be identified on the seismic lines and to be modelled was made based on the main aquifer and aquitard formations of the Paris Basin identified in the well reports (see the “Geological and Hydrogeological Contexts” section). Some of the connected aquifer or aquitard formations need to be grouped together to simplify the stratigraphy and due to seismic vertical resolution issues. Some formations, although identified in the wells (Table 2), are not thick enough to be visible on seismic (vertical resolution of approximately 20 m, see the “Horizon picking and interpretation” part of the “Methods” section below) nor to be considered as reservoir or aquitard formations (see the “Geological pile definition” part of the “Methods” section below). From bottom to top, the aquifer (A) and aquitard (a) formations are: the Basement (a), Triassic Sandstones (A), Liassic and Triassic Marls (a), Liassic Limestones (A), Toarcian Shale (a), Dogger Limestones (A), Callovo-Oxfordian Shale (a), Lusitanian Limestones (A), Kimmeridgian Marls (a) and the Tithonian Limestones (A) and the sandy lower Cretaceous (A). With regards to the faults, only the newly interpreted Sennely fault was modelled, because it is the only one that falls within the model extent.

### Seismic interpretation

Seismic reflection is a geophysical method for obtaining 2D images of the subsurface geological structures. There is contrast in rock properties between the different geological units where the acoustic waves bounce off, hence generating reflection. These surfaces are called “reflectors” and are represented by “reflections” on the seismic image. They are recorded in Two-Way-travel-Time (TWTT, usually s or ms), which refers to the travel time that takes the acoustic waves from the source to reflect on the interface and be received at the surface receiver.

#### Retrieve and reprocess raw data

Once retrieved, the seismic data was prepared to a numerical format to be reprocessed. The Loury-Villeny and Chartres seismic campaigns were acquired in the 1960s and therefore their raw data (recorded during the seismic acquisition) were recorded on analogic tapes. It was first necessary to transcribe them into a numeric format. The quality and consistency of the raw data was checked to ensure that the digitized seismic signal, the recording report and the source and receiver coordinates were consistent and to avoid an inadequate reprocessing and subsequent seismic imaging. Once checked, the seismic lines from the Loury-Villeny and Chartres datasets were reprocessed to convert the raw seismic data into a seismic image suitable for geological interpretation. The main objective is to obtain the best image of the sub-surface, given the vintage data available. Seismic reprocessing used modern signal-processing algorithms to significantly improve the quality of the images obtained in the 1960’s (see examples in the Paris Basin in Beccaletto *et al*.^39,46^ and Laurent *et al*.^47^). Several steps were necessary to obtain a satisfactory seismic image of the sub-surface. The first involved updating the acquisition geometry. It geographically positions the sources, receivers and seismic traces. A processing sequence is then applied that is comprised of spherical divergence compensation, velocity picking, noise attenuation, surface consistent deconvolution, surface consistent amplitude compensation, refraction and residual statics, stack and migration^48,49^.

Efforts were focused on three key steps that were repeated several times throughout the processing sequence: (a) the calculation of primary and residual static corrections in order to remove the topographic and velocity effects of the superficial rock layer, which strongly impact the seismic signal; (b) a detailed velocity analysis (migration); and (c) various methods of organized and random noise attenuation.

As a last step, the resulting reprocessed seismic lines were then exported in SEG-Y format - SEG-Y as this is the standard format for storing and using seismic data, read by all seismic processing and interpretation software^50^.

#### Import seismic reprocessed data and well data

The reprocessed seismic data were imported into the seismic interpretation module Geophysics in the GVERSE software^51^. The seismic lines from the Chartres and Loury-Villeny campaigns were merged with the Loiret 1982/Loiret 1983 lines by modifying their datum elevation. The vertical resolution of the reprocessed seismic lines is around 20 m.

The five wells were also integrated into the project in GVERSE’s Wellbase module to be used as a reference for the accurate depths and stratigraphy. Information from their survey report about their coordinates, head elevation, total well depth, formation top depths, deviation surveys and velocity surveys were entered. Since the seismic lines were in two-way travel time, it was necessary to convert the formation top depths to time. When a velocity survey was available (Table 1), it was also imported to convert the well to two-way travel time and thus, allow the seismic to well tie. Otherwise, the velocity survey of the nearest well was used.

#### Horizon picking and interpretation

**Fig. 4:**
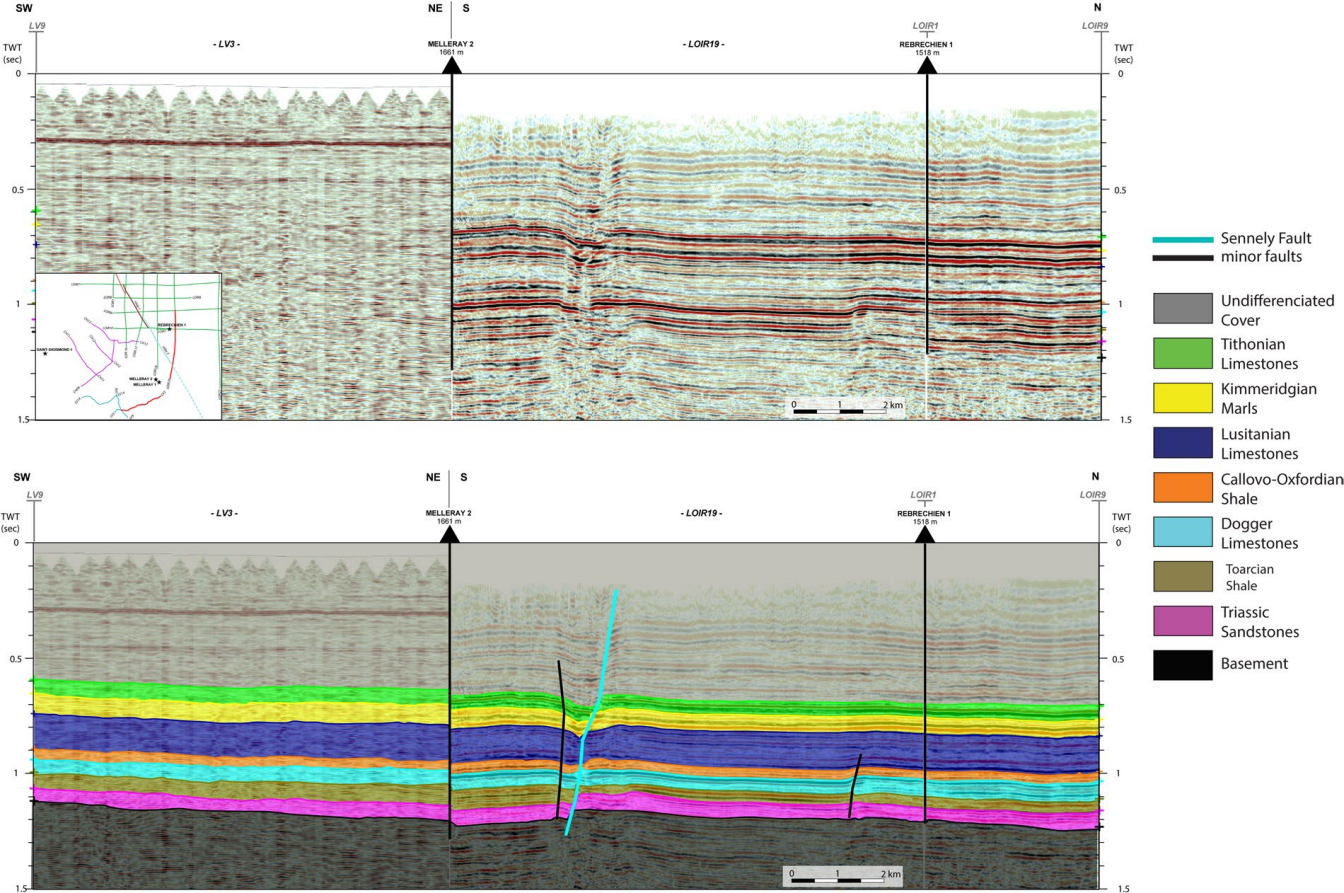
Snapshot of the composite seismic line LV03-LOIR19 showing the Sennely fault (blue), conjugate faults (black) and the projection of the Melleray 2 well on the composite line.

Seismic interpretation consists of recognizing seismic reflectors. The presence of nearby wells, converted in two-way travel time, (e.g. Rebréchien) makes it possible to match a formation top to a reflection, checking for consistency with the amplitude of the seismic signal, which provides information on the rock properties contrast. The reflection can then be tracked laterally and all the points of a picked reflection form a “seismic horizon” (Fig. 4). Reflections were associated with the formation tops selected for geomodelling (see the “Model setup” part of the “Methods” section above) (Fig. 4), from the base to the top: top Basement, top Triassic, top Lias (i.e, top Bathonian (i.e. Top Dogger Limestones), top Callovo-Oxfordian Marls, top Lusitanian Limestones (Oxfordian), top Kimmeridgian Marls, and top Tithonian Limestones.

Regarding the seismic to well tie and resolution issues, the geophysical properties of the rocks in the main aquifers and aquitards (and thus their reflections) are significant enough to discriminate them on the seismic lines despite their relatively low thickness. The seismic horizons are therefore clearly identified on the seismic lines with continuous and well-defined seismic facies.

The high density and regular spacing of the seismic lines in the western part of the model area, together with the relatively simple geological context (few faults, horizontal layers, well-defined geological formation, strong impedances contrasts), minimize the errors during the interpretation process

The horizons thus created provide information on the “depth” of the formation tops between wells in two-way travel time (TWTT), which are used for the OM3D geomodel development once converted into depths.

#### Fault identification and interpretation

The seismic interpretation has been used to define the geometry of the Sennely fault, which crosses the study area; it was identified on the seismic lines of the Loiret 1982/Loiret 1983 campaigns.

Since the Sennely fault is a relay fault^38^, some characteristics were studied on the seismic lines to identify the different segments: (1) the vertical extent, (2) the maximal apparent vertical offset, and (3) the approximate dip of the fault (not a real dip as the lines are in TWTT). With regards to the two former characteristics, the deformation field of a fault is an ellipsoid, the offset and the vertical extent are smaller at the extremities than in the middle of the fault segment. The apparent dip is assumed to be constant for a given segment.

It can be noticed that there are flexures near the fault between two seismic lines where the fractured rocks thickness and offsets are the lowest. These flexures are located on either one side of the fault or the other depending on the line. One hypothesis is that these flexures could be the ends of fault segments that appear respectively on the other line, forming a relay. The flexures are more developed at depth, meaning that the ends of the fault are anchored deeper.

**Fig. 5:**
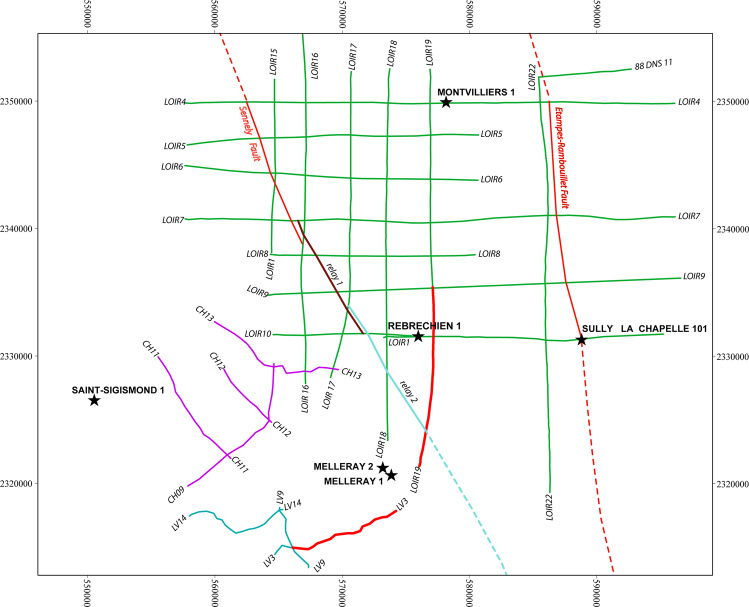
Map featuring the geometry of the Sennely fault following the interpretation in the relay fault. Note that the map is not centered around the OM3D geomodel domain (represented in blue). The RGF93/Lambert-93 coordinate system is used.

This case can be observed between lines LOIR7 and LOIR16 as well as between lines LOIR17 and LOIR10 (Fig. 5). Consequently, the fault was divided into three individual segments for the OM3D geomodel. The three segments of the fault have the same direction of 150° SE. Figure 5 also shows that the fault dip increases towards the south-east, which is consistent with the regional structural context.

Lastly, it can be noticed that the Sennely fault is not noticeable on all lines, meaning the offset is below seismic resolution of these lines. This is consistent with the aforementioned observations and the description of the relayed fault network provided by Héritier and Villemin^38^.

#### Velocity model and time-depth conversion

Seismic lines contain information in two-way travel time (TWTT) and the seismic horizons interpreted in time have therefore been converted to depth using a velocity model before being integrated into the 3D model. This is a key step, and it is directly dependent on the seismic velocities in the underground.

Since there was no velocity survey for each well, the velocity model was established by first combining the depth of the formation tops in the wells and the horizon picking in two-way travel time in order to obtain a velocity value per formation at the well location. These local velocity values were then interpolated throughout the whole area (minimum curvature algorithm, cells spanning 250 m^2^), resulting in a velocity model with varying velocities for a given geological interval.

We used interval velocities rather than average velocities, as is usually done in the Paris Basin when depth-converted seismic horizons are the input data for 3D modelling (its advantage is that it avoids the converted horizons from crossing each other^52,53^).

Generally speaking, the interval velocity of a given formation corresponds to the mean velocity within this formation:

$${V}_{int}=\frac{{Z}_{base}-{Z}_{top}}{OW{T}_{base}-OW{T}_{top}}$$

where OWT = one-way time, Z = depth.

As a result, we considered every geological formation of the stratigraphic pile of the model as a distinct layer with a varying interval velocity in the entire study area. This calculation was performed in the GVERSE Geophysics software. Velocities mostly range from approximately 2000 m/s to approximately 4000 m/s for all of the formations, which are typical values in this area of the Paris Basin^35^. For each formation, the thickness values for the interval between the current and previous horizon were calculated using the velocity values associated with that interval. The final depths were calculated by successively adding the thicknesses of the formation calculated in this way. The interpreted seismic horizons have then been converted to depth step by step from the shallowest (Top Tithonian) to the deepest one (Top Basement).

Like any other TD conversion method, this interval velocity conversion method has some limitations. Because the interval velocities are first calculated based on data from five boreholes (before being interpolated at the scale of the 3D model), the TD conversion of the seismic horizons does not consider the potential lateral variations of lithologies and rock properties. Therefore, the uncertainties increase as the distance from the reference boreholes increases. However, given the similar lithologies for each layer (as described in the boreholes and from regional geology, e.g. Guillocheau *et al*.^29^, Lenoir *et al*.^35^), the facies variations are very likely not significant enough at the regional scale to have a major impact on the velocity behavior of the different units.

At the end of the process, a selection of data points (X, Y, Z in meters) along the seismic lines of all the depth converted horizons were exported from GVERSE Geophysics in a table format, ready to use in GeoModeller. On average, the spacing of the points in X,Y is 800 m.

### Data preparation and import for geomodelling

#### Digital terrain model

Once the domain was defined, the BD ALTI® digital terrain model (IGN) was imported into GeoModeller to constrain and form the topographic surface of the OM3D geomodel.

#### Geological pile definition

**Fig. 6:**
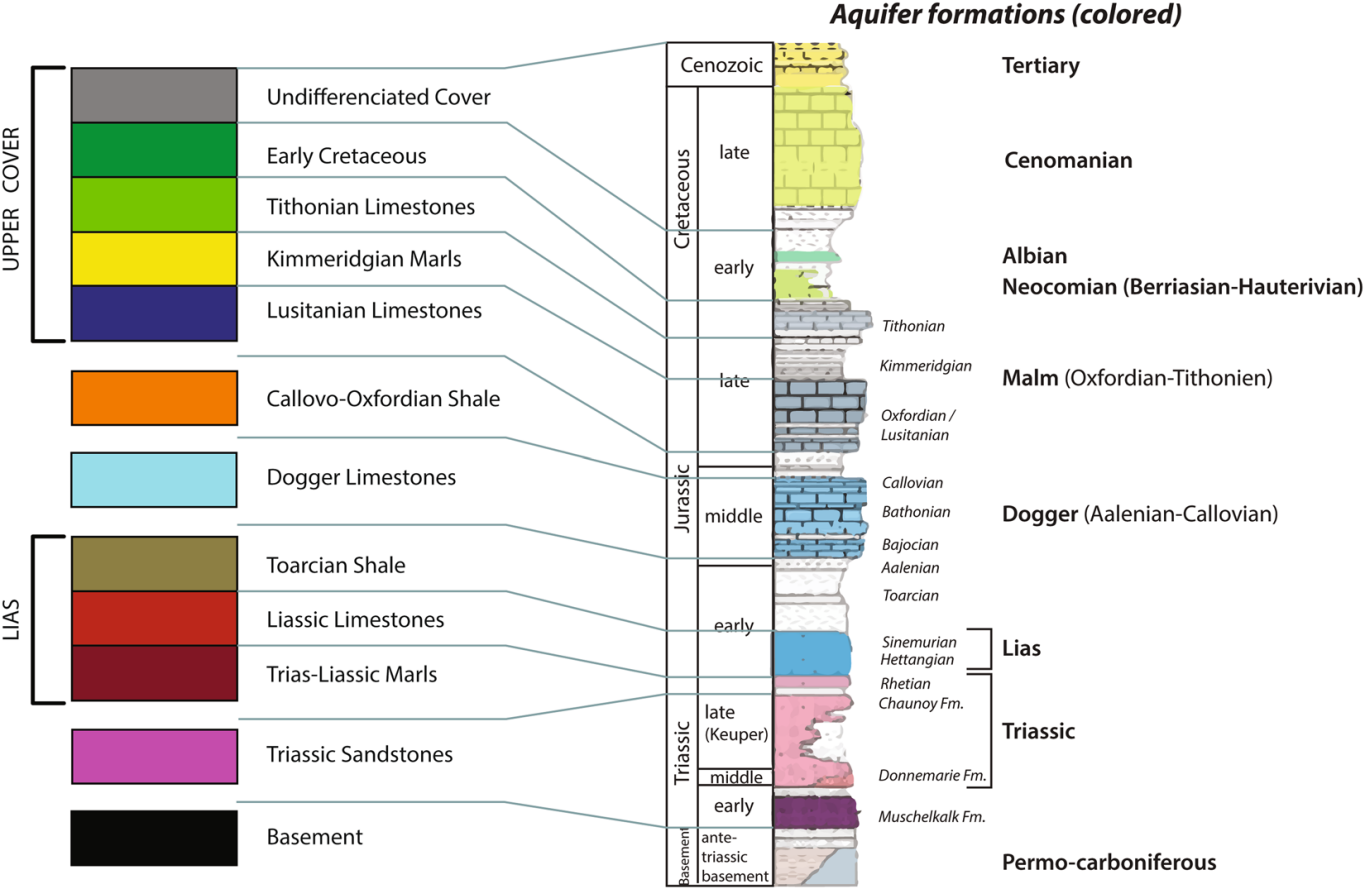
Geological pile defined for the OM3D geomodel development and the corresponding levels on the stratigraphical log of the Paris Basin with the major aquifer (colored) and aquitard (uncolored) formations (modified after Delmas *et al*.^32^). Some formations were grouped together to form “series” to identify aquifers and aquitards.

The “geological pile” refers to the sequence of lithological formations and allows an automatic management of the relationships between the geological bodies during the geomodelling process (see the “Code Availability” section). Several individual formations can be grouped together, forming a “series”^54^. The geological pile and series were composed for a hydrogeological purpose (see the “Model setup” part of the “Methods” section above). Consequently, the formations were grouped together according to the main aquifers and aquitards. Formations at the top were grouped to form the “Undifferentiated Cover”, because they cover the aquitard lying over the upper targeted reservoir and do not need to be differentiated. The final geological pile is shown in Fig. 6.

#### Well data

Well data were imported in GeoModeller from the spreadsheet containing information about their coordinates, formation names, and depths of the formation tops. These pieces of information were recovered from the borehole survey reports.

#### Geological map and sennely fault trace

The georeferenced geological map with the Sennely fault trace (Fig. 3) was imported into GeoModeller and wedged in the topographic section.

#### Pre-existing larger-scale lower-resolution 3D geological model

The surfaces of the pre-existing larger-scale lower-resolution 3D geological model corresponding to the formation tops to be modelled were integrated in the GeoModeller project.

#### Seismic horizons data

**Fig. 7:**
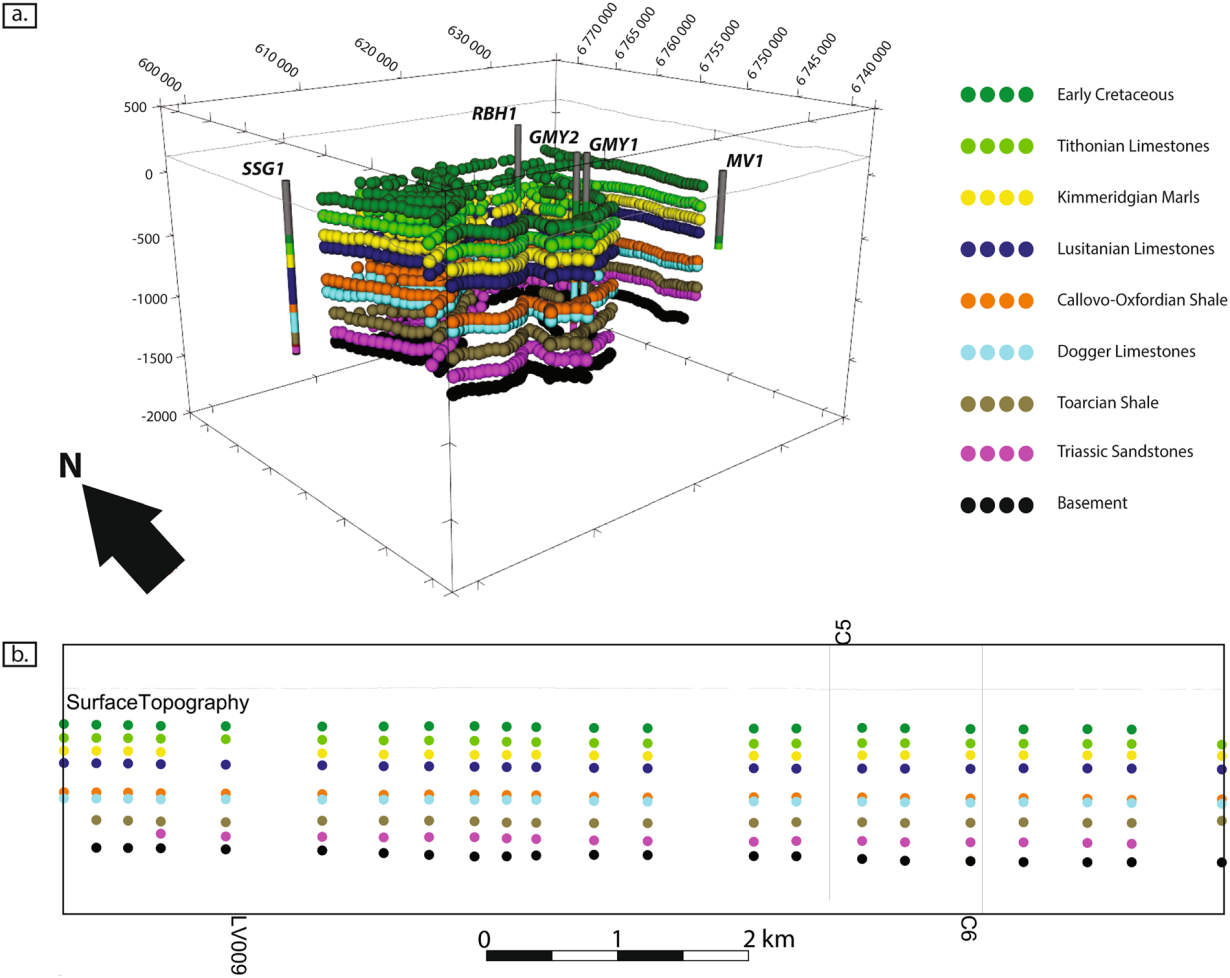
(**a**) 3D view of the horizon picking points integrated in GeoModeller (note that the formations of the undifferentiated cover are not shown), (**b**) 2D view of the horizon picking points projected in the LV03 cross-section (Fig. 3; note that the formations of the undifferentiated cover series are shown). The RGF93/Lambert-93 coordinate system is used.

In GeoModeller, the seismic data import involves two steps: the broken-line cross-sections corresponding to the seismic lines were first created using their coordinates, and the picking points of the time-depth converted horizons were then integrated into the 3D software (Fig. 7).

During import, each horizon was associated with the corresponding formation of the previously created geological pile to constrain the interpolation of the formation’s geometry.

#### Seismic images and digitalization of fault interfaces

**Fig. 8:**
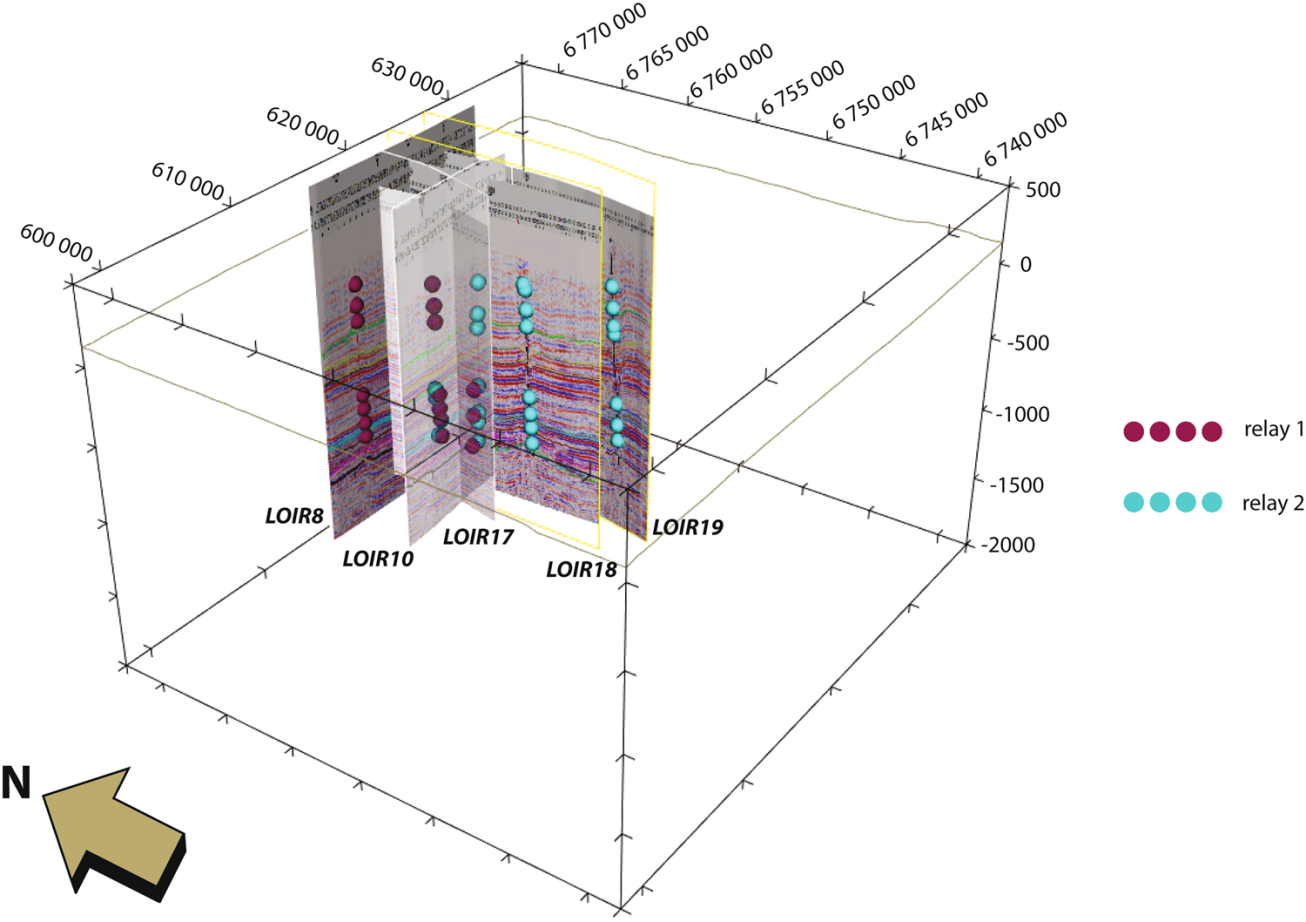
3D view showing the projected seismic lines in the cross-sections and the digitized points associated with the Sennely fault. The blue and red dots show the location of the two segments of the fault. The RGF93/Lambert-93 coordinate system is used.

Aside from the tops of the formations, the Sennely fault is the second type of geological object to be modelled. The GVERSE Geophysics software does not enable the fault points to be exported because the time-depth conversion cannot be applied to this type of geological features. It was therefore necessary to adopt another approach to integrate these data into the GeoModeller in two steps: (1) georeferencing and vertical stretching of the seismic image: each seismic line including a trace of the Sennely fault was entered as a screen shot in the seismic interpretation software and then georeferenced. The key problem was to match the images of the seismic lines shot in two-way time within the OM3D geomodel cross-sections in real depth. This was accomplished using the previously integrated horizon points: interpreted seismic line images were vertically adjusted to match the horizon points projected in the cross-sections. (2) digitizing the subsurface fault interfaces: once the seismic line images were fitted to the horizons, the fault lines were digitized (Fig. 8) and the orientation data were placed according to the fault trace on the image to better constrain the fault geometry. However, the image adjustment does not allow for a perfect match of every horizon in a given cross-section. To ensure optimal stretching, a seismic line image was fitted horizon by horizon, involving several calibrations per line.

To the south-west of the OM3D geomodel, where there is no seismic data, contact points and orientation data were given by the Sennely fault digitized following Beccaletto *et al*.^40^. These data points were added to the topographic section.

### 3D geomodel processing

The potential field method implemented in GeoModeller was used to interpolate the OM3D geomodel. This method allows to infer the geological formation for any 3D points (x,y,z), and produces a continuous geomodel (see Calcagno *et al*.^54^ and the “Code Availability” section).

The sedimentary filling of the area to the south-west of the Paris Basin is relatively continuous, and the geological data of the Orléans region did not reveal any major discontinuity. Consequently, the ‘Onlap’ relationship was assigned to every series in the geological pile, meaning that a given series simply ‘onlaps onto’ the older ones (see the “Usage Notes” section). Likewise, the links between the Sennely fault and geological series were defined to state that the fault affects the whole series, as observed on the seismic lines. Moreover, the two segments of the Sennely fault included in the OM3D geomodel’s domain were modelled by two finite faults to obtain a reliable representation of the relay: finite fault boundaries are modelled as elliptic surfaces, since the displacement decreases towards its limits (see Calcagno *et* *al*.^54^ for more information). The radius of the ellipsoids was defined to match the seismic interpretation (see Fig. 5 and the “Fault identification and interpretation” part of the “Methods” section above), i.e. the extent was adjusted so that each fault segment crosses only the seismic lines where it has been observed. The parameters used in GeoModeller to constrain the finite faults are given in the Table 4.

**Table 4:** Parameters used to model the two finite faults representing the Sennely fault relay located in the OM3D geomodel extent.

	Relay 1	Relay 2
Horizontal Radius (m)	7500	26000
Vertical Radius (m)	1500	2500
Influence Radius (m)	1500	5000
Center	Mean Center	User specified (x = 632133.5; Y = 6749049.102; Z = −1700)

The seismic and wells data used for the construction of the OM3D geomodel are mostly located in the north-east and in the center of the study area. Elsewhere, the formations architecture and fault geometry were constrained by the pre-existing larger-scale and lower-resolution 3D geological model: eight additional cross-sections were constructed for this purpose and additional control points were added to them. In the south-east of the OM3D geomodel, it was completed by the revised cartographic data for the delineation of the Sennely fault (see the “Geological map” part of the “Input Data” section above).

### Export

**Fig. 9:**
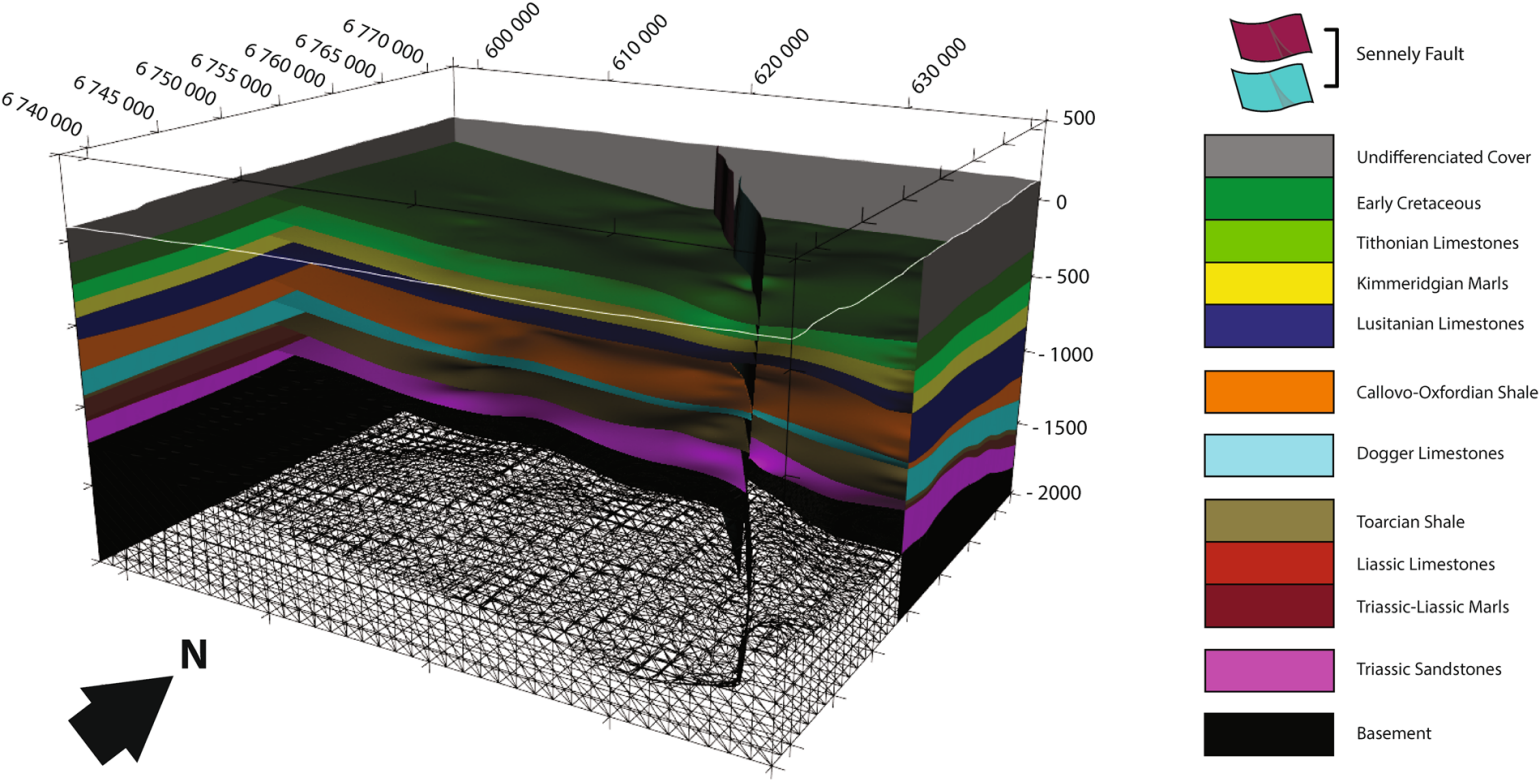
View from the southwest of the OM3D geomodel in the 3D Window of GeoModeller. The RGF93/Lambert-93 coordinate system is used.

**Fig. 10:**
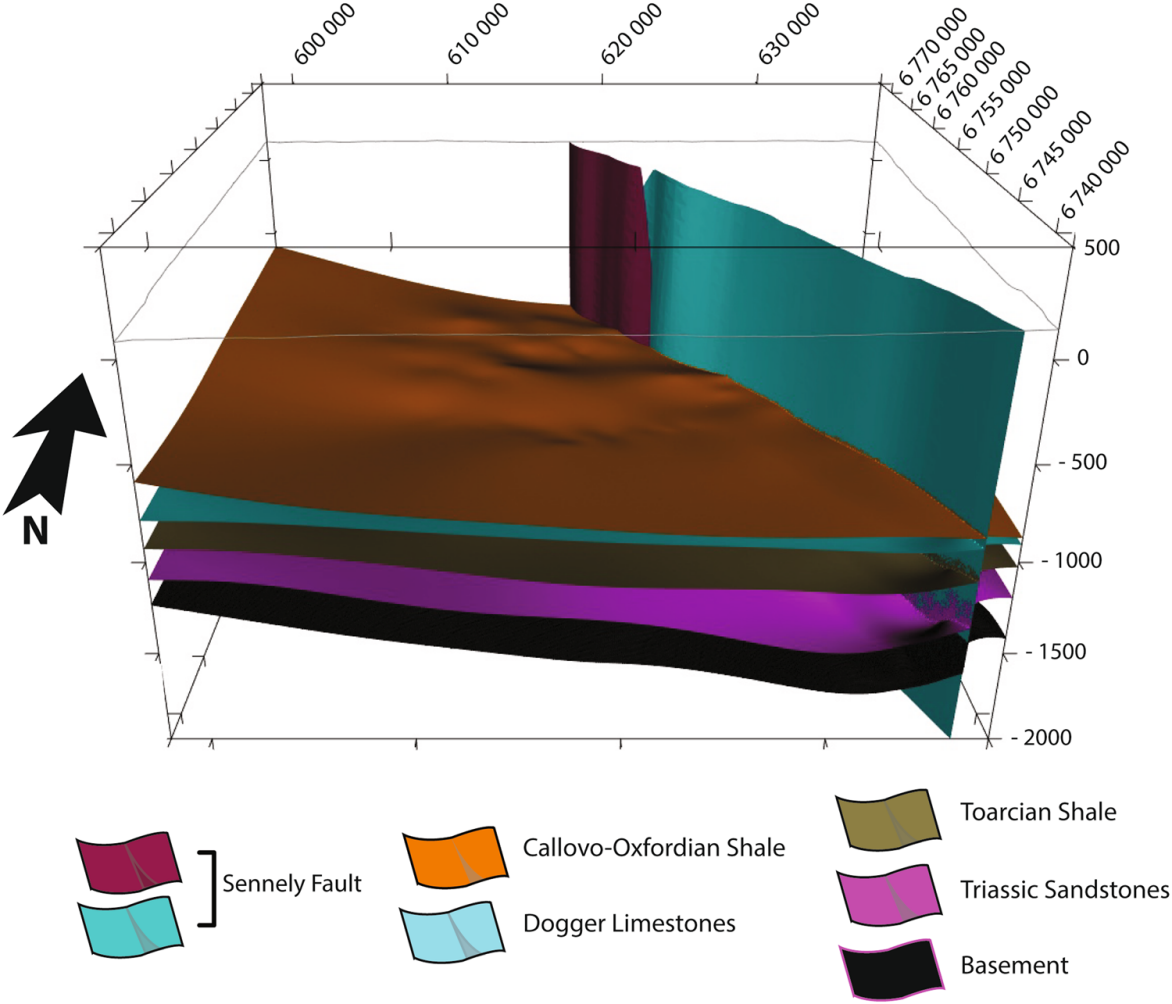
View from the south of the OM3D geomodel in the 3D Window of GeoModeller showing the main aquifer formation tops; note the relay geometry of the subvertical Sennely fault. The top right inset is the topographic section of the geomodel displaying the geological map and the modelled Sennely fault on the surface. The RGF93/Lambert-93 coordinate system is used.

Once it was determined that the OM3D geomodel is an acceptable representation of reality (see the “Technical Validation” section), it was saved in its native format and can thus be opened, visualized, or modified directly in this software (Figs. 9,10). Since GeoModeller also has geophysical and geothermal modules, further modelling processes can be performed in the software.

In addition, the OM3D geomodel was exported in several formats (see the “Usage Notes” section) with a grid cell resolution of 100 m × 100 m × 10 m. These export formats can be used as base models for further modelling or simulations. A 3D PDF was produced. This format is very practical because it can be opened and accessed from most PDF reader softwares. The geomodel can be manipulated, and the geological volumes and interfaces can be shown or hidden. Therefore, this format is useful for communication purposes.

3D shapes were also exported in two format types: the first one is the TSurf format that can be imported in many modeling or simulation softwares, and the second one is the ParaView VTK (i.e. Visualization ToolKit) Poly Data format that can be visualized in the open-source 3D visualization software ParaView. The formation interface surfaces can be loaded as polygonal meshes and processed by the ParaView tools.

## Data Records

The OM3D geomodel^55^ is recorded in the Zenodo repository under the Creative Commons Attribution 4.0 International (CC BY 4.0) license. The geomodel comes with a metadata sheet in pdf format which summarizes the technical parameters related to the model such as location, description, and contact.

The OM3D geomodel is made of 11 formations in total, grouped in six series onlapping on top of each other. Two finite faults represent the two segments of the Sennely fault crossing the study area and forming a relay (Fig. 10).

The OM3D geomodel should be considered as a “*data augmentation exercise*” as proposed by Thornton *et al*.^24^. It shows how a multi-disciplinary integration allows for the knowledge of the subsurface to be refined. This work delivers a geomodel that is particularly suitable at the scale of the metropolis that can be used as a base for various future calculations, such as hydrothermal simulations. It would enable the metropolis to have a representation of its subsurface that can be updated when new data are acquired.

## Technical Validation

Several considerations were taken into account during the seismic interpretation and the modelling steps to ensure the reliability of the OM3D geomodel: the quality of the input data, the methodology used for the modelling and the continuous checking of the geological plausibility of the results.

First, most of the input data, seismic lines, and boreholes, were acquired by petroleum companies between the 1960s and the 1980s. These data are particularly suitable for the identification and modelling of the main aquifers of the Orléans metropolis’ area as these companies were targeting mostly the same reservoirs. Moreover, the reprocessing techniques used for this study have improved the quality of the resulting seismic images, making the identification of the geological interfaces easier and more accurate compared to the original processing.

The wells are located on (or very close to) the seismic lines, and some of them have velocity surveys. This is very important (1) to display the wells in depth on the seismic lines in TWTT, and (2) to ensure the accuracy of the horizon’s location in real depth for the time to depth conversion. Moreover, the geological context is very appropriate for the depth-conversion process: the layers are quite tabular and horizontal, and the structural aspect is relatively simple. Those conditions improve the reliability of the time to depth conversion^56^. The interval velocity method has also been chosen as the most robust one to ensure the reliability of the horizons converted to depth. A quality control has been performed when importing the well and seismic data in the OM3D geomodel to compare the depths of the formation tops in the wells with the resulting horizons converted in depth.

Furthermore, the approach and methodology used to create the OM3D geomodel helped improve its reliability: many independent data types and datasets were integrated such as well data, seismic data, a Digital Terrain Model (DTM) and a pre-existing larger-scale and lower-resolution 3D geological model. The crossing of these data improves the consistency and reliability of the OM3D geomodel.

Regarding the validity and accuracy of the OM3D geomodel calculation by the GeoModeller software, it has been demonstrated that its algorithm correctly takes the input data into account^24,57,58^. Consequently, the objects modelled using this methodology give a realistic description of the geology, in accordance with the input data and without creating uncertainties.

This is also ensured by the geological expertise of the operators during the modelling process, who can validate or not the results and the geomodel plausibility based on their knowledge of the geological features represented. As a consequence, the modelling of the Sennely fault is in accordance with the state of knowledge on its geometry^38,40^.

**Fig. 11:**
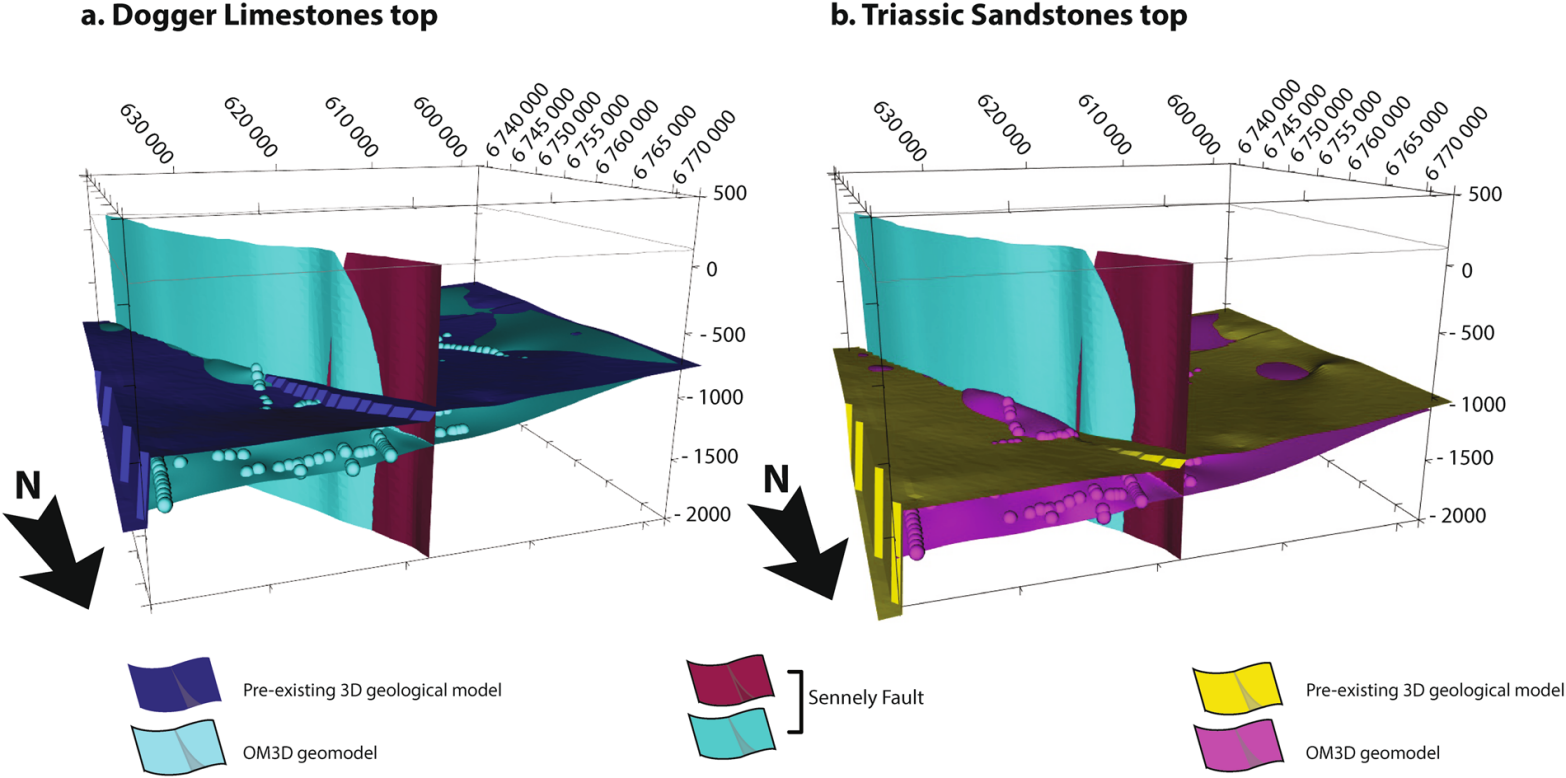
Comparison between the surfaces of the Dogger Limestones (left) and the Triassic Sandstones (right) extracted from the OM3D geomodel and the pre-existing larger-scale lower-resolution 3D geological model. The dots represent the horizon picking points of the formation tops. The RGF93/Lambert-93 coordinate system is used.

As a result of this interdisciplinary methodology, the OM3D geomodel highlights the added value of the use of seismic data.The surfaces of the Dogger and the Triassic tops of the OM3D geomodel are better constrained thanks to the seismic data which improve the location of these geological interfaces. They appear deeper than those of the pre-existing larger-scale and lower-resolution 3D geological model in the north-east corner which is, as a reminder, the only one covering this study area and with a larger scale. This difference can reach 500 m in depth but is very local (Fig. 11). Another improvement of the OM3D geomodel concerns the northern part of the Sennely fault. From a structural point of view, the OM3D geomodel gives a more detailed representation of the Sennely fault with a better-defined geometry which is also consistent with the geological reality of late Variscan faults (Fig. 11).

In another validation phase, the OM3D geomodel was compared with a gravimetric survey. Gravimetry is a geophysical method for detecting variations in gravity, which can be related to variations in the density of deposits. Negative gravimetric anomalies represent density deficits, corresponding to cavities or lighter geological formations. Positive gravimetric anomalies represent excesses of density, corresponding to the presence of minerals, faults or massive terrain rising close to the surface.

**Fig. 12:**
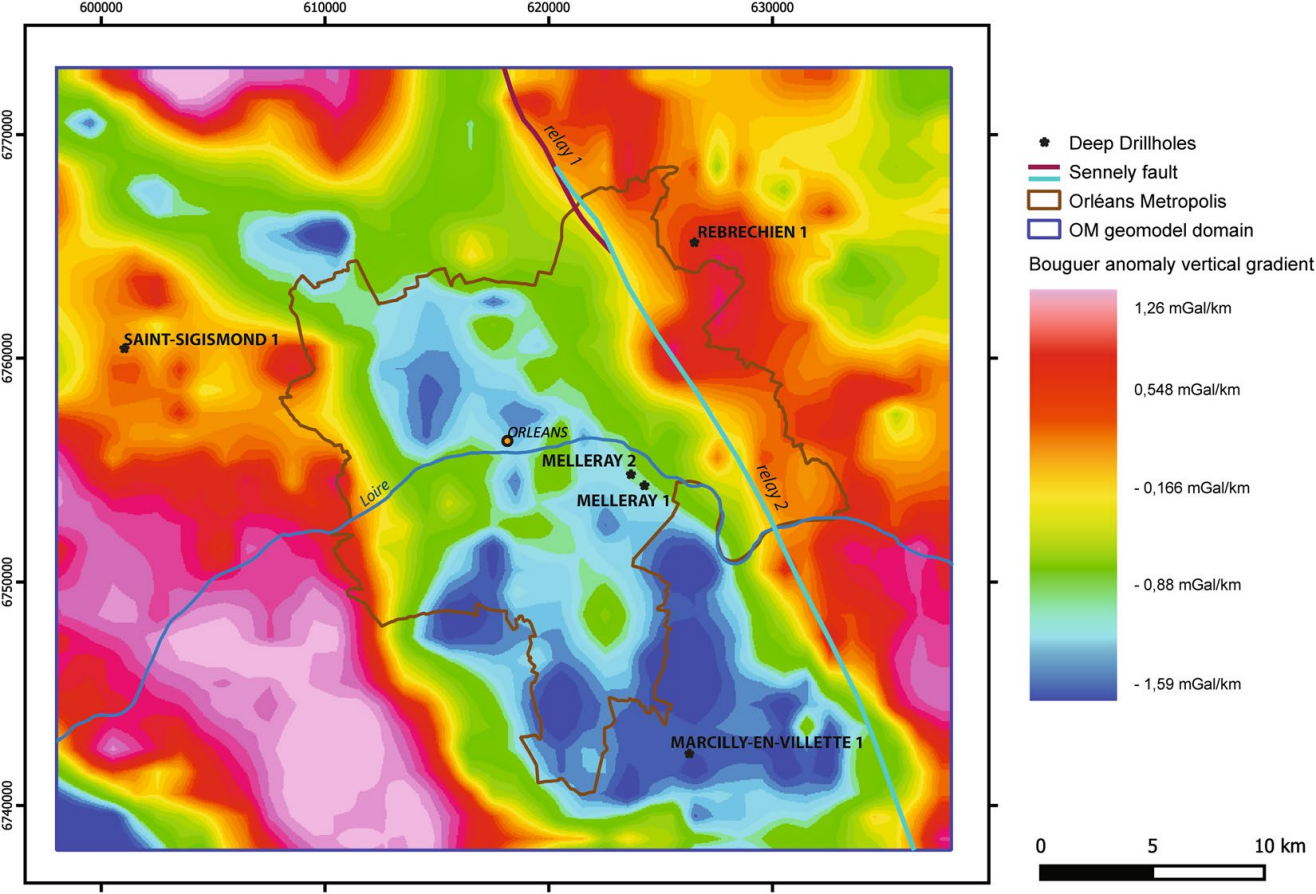
The gravimetric map shows a graben structure to the south-west of the modelled area, in accordance with the structure in the OM3D geomodel. Modified after Martelet *et al*.^59^. The Sennely fault trace displayed on the map originates from a section of the OM3D geomodel at a depth of 1000 m.

A vertical gradient of the vertical anomaly map from Martelet *et al*.^59^ (Fig. 12) shows negative anomalies to the south-east of the modelled area which could be explained by both the deepening of the basement and the over-thickness of the overlying sedimentary series in relation to the westward downthrow of the Sennely normal fault offset^27^. From a structural point of view, this can be interpreted as a regional horst and graben structure: the NW-SE axis horst is delimited between the Sennely and Etampes-Rambouillet faults and a semi-graben is located to the west of the Sennely fault.

A sedimentary thickening is present in the south-west of the OM3D geomodel, in accordance with the gravimetric map (Figs. 9,10). Even if the gravimetric map and the OM3D geomodel give a representation of two different objects (the basement for the former and the sedimentary cover for the latter), they show similar trends and features, validating the accuracy of the OM3D geomodel at a large scale.

Some challenges were encountered during the development of the OM3D geomodel. Its limitations are mainly based on the distribution and density of the primary data: there are fewer seismic or borehole data in the north-west, south-west and south-east corners of the OM3D geomodel and within the metropole; as a consequence, a pre-existing larger-scale lower-resolution 3D geological model, which is a result of a prior interpretation work, was used to constrain the OM3D geomodel. Lastly, the ideal technical validation would be new drilling operations to check and assess the reliability and specify the OM3D geomodel since no data have been withheld, as discussed in Thornton *et al*.^24^.

## Usage Notes

### Formats

The geomodel available in the Zenodo repository is in the original GeoModeller format, PDF 3D, TSurf, and VTK. The file formats can be read using the tools listed in Table 5.

**Table 5:** OM3D geomodel file formats available on the Zenodo repository and software to read them.

File format	Software
GeoModeller	GeoModeller, https://www.geomodeller.com
PDF 3D	Standard Adobe pdf reader, https://get.adobe.com/fr/reader/
TSurf	GocadTSurfaceReader, https://www.opengeosys.org/docs/tools/fileio/gocadtsurfacereader/
VTK	Paraview, https://www.paraview.org/download/

## Data Availability

**GeoModeller** GeoModeller is a proprietary software developed by the French Geological Survey (BRGM) in collaboration with Intrepid Geophysics. It has been designed for three-dimensional interpretation and modelling based on the integration of various types of data, such as field observations and geophysical measurements. The calculation of the model is based on geological rules. These rules are defined in a geological pile that manages the relationships between the geological formations to be modelled^54^. Data are associated with formations and faults to constrain their geometry. The modelling of these geological objects relies on the interpolation of the input data. GeoModeller’s interpolation method produces a scalar field where iso-values represent the geological interfaces. The scalar field is interpolated by co-kriging two types of data: (1) 3D points that are the location of observed or interpreted geological interfaces and faults, and (2) orientation data that are 3D vectors representing the dip of geological formations. These two types of data are respectively associated with the iso-values and the gradients of the scalar field to be interpolated^54,60^. GeoModeller also enables direct and inverse geophysical calculations to improve and refine the geomodels^61^. For further information, see: https://www.geomodeller.com The GeoModeller version used for this study is the following: GeoModeller Version: 4.0.7 Build Date: May 22, 2019 Build Number: 27eee3dc31ba The default parameters of GeoModeller were used for the interpolation of the geomodel presented in this paper.
